# EEG-Based Sleep Staging Analysis with Functional Connectivity

**DOI:** 10.3390/s21061988

**Published:** 2021-03-11

**Authors:** Hui Huang, Jianhai Zhang, Li Zhu, Jiajia Tang, Guang Lin, Wanzeng Kong, Xu Lei, Lei Zhu

**Affiliations:** 1 School of Computer Science and Technology, Hangzhou Dianzi University, Hangzhou 310018, China; Hyoui7890@gmail.com (H.H.); jhzhang@hdu.edu.cn (J.Z.); hdutangjiajia@163.com (J.T.); lindandan@hdu.edu.cn (G.L.); 2 Key Laboratory of Brain Machine Collaborative Intelligence of Zhejiang Province, Hangzhou Dianzi University, Hangzhou 310018, China; kongwanzeng@hdu.edu.cn; 3 Sleep and NeuroImaging Center, Faculty of Psychology, Southwest University, Chongqing 400715, China; xlei@swu.edu.cn; 4 Key Laboratory of Cognition and Personality, Ministry of Education, Chongqing 400715, China; 5 School of Automation, Hangzhou Dianzi University, Hangzhou 310018, China; zhulei@hdu.edu.cn

**Keywords:** sleep staging, electroencephalography (EEG), brain functional connectivity, frequency band fusion, phase-locked value (PLV)

## Abstract

Sleep staging is important in sleep research since it is the basis for sleep evaluation and disease diagnosis. Related works have acquired many desirable outcomes. However, most of current studies focus on time-domain or frequency-domain measures as classification features using single or very few channels, which only obtain the local features but ignore the global information exchanging between different brain regions. Meanwhile, brain functional connectivity is considered to be closely related to brain activity and can be used to study the interaction relationship between brain areas. To explore the electroencephalography (EEG)-based brain mechanisms of sleep stages through functional connectivity, especially from different frequency bands, we applied phase-locked value (PLV) to build the functional connectivity network and analyze the brain interaction during sleep stages for different frequency bands. Then, we performed the feature-level, decision-level and hybrid fusion methods to discuss the performance of different frequency bands for sleep stages. The results show that (1) PLV increases in the lower frequency band (delta and alpha bands) and vice versa during different stages of non-rapid eye movement (NREM); (2) alpha band shows a better discriminative ability for sleeping stages; (3) the classification accuracy of feature-level fusion (six frequency bands) reaches 96.91% and 96.14% for intra-subject and inter-subjects respectively, which outperforms decision-level and hybrid fusion methods.

## 1. Introduction

With social pressure increasing in this high-speed development era, more and more people are faced with deep sleeping problems. The chronic lack of sleep or getting poor-quality sleep is a risk factor for cognitive disorders, mood disorders, and diseases such as high blood pressure, cardiovascular disease, diabetes, depression, and obesity [1,2]. Sleep staging is the basis of sleep quality evaluation, and plays an important role in the early diagnosis and intervention of sleep disorders. In 1968, R&K [3] identified sleep staging into awake, rapid eye movement (REM) and non-rapid eye movement (NREM) stages, and NREM is further subdivided into four stages: S1, S2, S3, and S4. Since S3 and S4 are similar in many aspects, American Academy of Sleep Medicine (AASM) [4] revised the R&K rules and used N1, N2, N3 to represent different sub-stages for NREM stage, combining both S3 and S4 into N3 stage.

In practice, clinical sleep staging is still based on visual inspection by sleep experts for decades according to the duration and proportion of special brain waves. Such waves during sleep include delta waves, alpha waves, sleep spindle waves and K-complex waves. Delta waves are slow waves, mainly appearing in N2 and N3 stages with different proportions. The frequency range of the alpha wave (8–13 Hz) and sleep spindle wave (12.5–15.5 Hz) is partially overlapping. Alpha waves generally appears in the REM stage. Both frequency band range and the occurring brain area of sleep spindle waves are different between N2 and N3 stage. The k-complex waves are the combination of apical waves and sleep spindle waves. The types and spatial distributions of these waves are different during sleep stages, see Table 1 for more details. Therefore, frequency bands should be considered in sleep staging analysis.

However, such manual sleep staging judgment by sleep experts easily brings problems of low efficiency, long time consuming, and subjective errors. Chapotot et al. [5] show that the average same judgment accuracy between two experts in labeling sleep-wake stage scores is only about 83%. Therefore, a more accurate and objective method for sleep staging is very required. Moreover, sleep is a complex and dynamic process, so that humans always hope to have a better understand of the brain mechanisms of sleep for human health. With the help of the signal recording technology, several sleep physiological signals acquisition methods are existed. For instance, polysomnography is a powerful tool for sleep signal acquisition including electroencephalography (EEG), electromyography (EMG), functional magnetic resonance imaging (fMRI), and electrooculography (EOG). Herein, EEG has advantages of low cost, high temporal resolution and easy operation which result in the wide application in sleep stages research [6,7]. Afterwards, sleep-related researchers take use of the recorded signals to conduct sleep staging research.

For computational sleep staging research, the main objective of this area is to find out discriminative features and good-performing classification strategies. Currently, EEG-based sleep staging research has brought out many desirable results such as most of the features are extracted from the single channel and end-to-end classifier models [8,9,10,11,12,13,14]. For instance, Ahmed et al. [15] designed a 34-layer deep residual neural network to classify the raw single-channel EEG sleep staging data and obtained the improved accuracy of 6.3%. This end-to-end classifier usually has a good classification performance, but it lacks the exploration of sleep mechanisms. On the other hand, Thiago et al. [16] proposed a feature extraction method based on wavelet domain, which increases the classification performance nearly 25% compared to the temporal and frequency domains; Zhang et al. [17] proposed a feature selection method based on metric learning to find out the optimal features. However, existing feature extraction methods (temporal, frequency and temporal-frequency domains) are difficult to explore the sleep staging information from a global level [18,19,20] since the calculation is performed on single-channel separately. [21,22] also pointed out that the amount of information obtained through a single channel does not fully characterize the changes in brain activity during sleep.

Brain functional network is a relative new measurement to characterize the information exchanging between brain region through calculating the temporal correlation or coherence between brain areas. It is verified that each sleep stage is associated with a specific functional connectivity pattern in fMRI studies [23,24,25]. EEG-based brain functional connectivity has been employed in sleep research [26,27,28] to distinguish the sleep disease and health groups. We would use functional connectivity to explore the synchronization mechanisms between different brain regions and the classification accuracy for sleep staging.

In summary, in addition to pursuing the higher classification accuracy, we also want to, within different frequency bands, explore the information exchanging between brain areas for sleep staging. Specifically, this paper analyzed the sleep stage with single-band functional connectivity and then used the bi-serial correlation coefficient method to evaluate the frequency bands. Based on the evaluation results, features from frequency band are fused at the feature-level, decision-level and hybrid-level, respectively. Furthermore, we also investigated the mutual influence between frequency bands to identify the sleep stages. The remaining parts of this paper are organized as follows. Section 2 describes the materials and our method. Section 3 indicates all results. Section 4 and Section 5 provides the discussions and summarizes the future work, respectively.

## 2. Materials and Methods

### 2.1. Dataset Description

**Figure 1:**
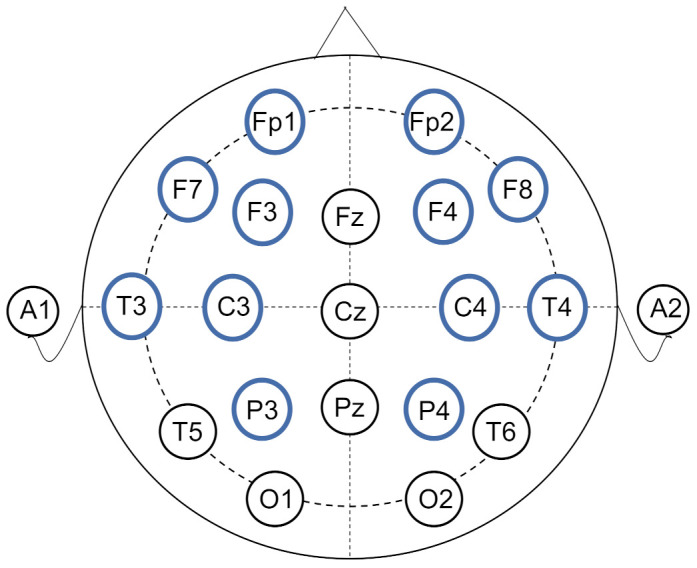
The layouts of EEG electrodes. The electrodes used in this study are labeled in blue circles.

The data analyzed in this manuscript is from the public CAP Sleep Database [29,30]. The database was built to facilitate sleep research that includes 108 polysomnographic recordings provided by the Sleep Disorders Center of the Ospedale Maggiore of Parma, Italy. There are 16 healthy subjects without any neurological disorders and drug problems. The number of EEG channels varies from 3 to 12 and the data with the number of channels as more as possible are needed for functional brain connectivity calculation, therefore, we selected the subjects (namely n3, n5, n10, n11 respectively) who were recorded with 12 EEG channels, aging between 23 and 35 (mean 30.25) years old. According to the International 10–20 System, the placements of the bipolar electrodes were Fp2-F4, F4-C4, C4-P4, P4-O2, F8-T4, T4-T6, Fp1-F3, F3-C3, C3-P3, P3-O1, F7-T3, and T3-T5, shown in Figure 1. The sampling rate is set at 512 Hz. For each subject, the continuous recorded sleep EEG lasted about 9 h (from 10:30 p.m. to 7:30 a.m.).

The experts labeled sleep stages based on the standard rules by R&K every 30 s and sleep is a cyclical process, the duration of a cycle is about 90 to 110 min, humans generally experience 4 to 5 sleep cycles per night [3]. Since the N1 accounts for 5–10% or less of the total sleep duration (only lasts about 1–7 min) [31], we selected the other three sleep stages including the REM and N2, N3 during non-REM.

### 2.2. Framework of Our Method

**Figure 2:**
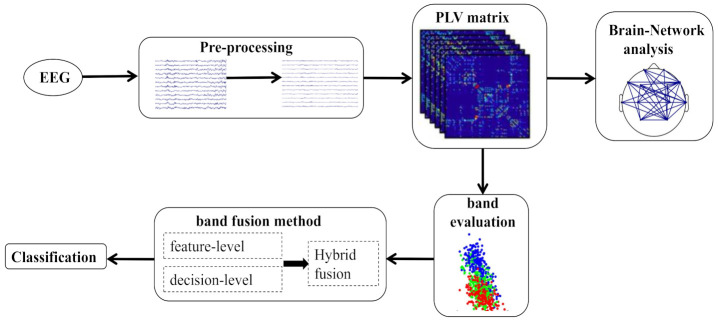
The flow diagram of the proposed method.

In this study, we first perform data preprocessing, then calculated the brain functional connectivity for different frequency bands to compare the characteristics of brain mechanism during sleep stages. Moreover, bi-serial correlation coefficient was adopted to evaluate brain connectivity features across different frequency bands. Then, we used three fusion strategies for frequency band fusion to classify the sleep stages based on the findings in the frequency band evaluation results. The framework of our method was depicted in Figure 2.

#### 2.2.1. Data Preprocessing

It is difficult to draw clear boundaries between different sleep stages because the stage usually changes gradually and continuously. To confirm the sampling with the exact sleeping stage label, we delete the following three kinds of data that:

- belonging to the same stage, but duration is too short (such as only 2 to 3 min).
- the unusual waking duration (tens of seconds) and its before and after 30 s duration during a certain sleep stage.
- the beginning 30 s and the last 30 s of a certain sleep stage.

After that, we segmented the EEG into 30 s epochs as analyzed samples without overlapping. Note: for sleep staging, the adopted 30 s epoch is derived from the R&K and AASM rules [32], and related works also revealed that 30 s length of epoch is viable to characterize intrinsic brain activity [33,34]. The total number of samples with sleep staging labels REM, N2 and N3 is *801*, 900, and 1001, respectively. Herein, the number of samples from subjects n3, n5, n10, n11 is *651*, 639, 559 and 853, respectively.

For data preprocessing, we adopted common average reference (CAR) [35] to minimize the uncorrelated noise among channels, then we removed the artifacts with independent component analysis (ICA) [36] and Adjust plugin which is realized in EEGLAB [37] followed by the band-pass filtering with a passband from 0.5 to 40 Hz. Furthermore, we filtered the denoised EEG into six frequency bands: delta (0.5–4 Hz), theta (4–8 Hz), alpha (8–13 Hz), beta1 (13–22 Hz), beta2 (22–30 Hz), and gamma (30–40 Hz).

#### 2.2.2. Phase-Locked Value

We estimated brain functional connectivity with phase-locked value (PLV). The PLV [38] was proposed to measure the phase synchronization between two signals which is only sensitive to phase but not to amplitude. Compared with other synchronization measures, PLV is simple to operate and can maintain the same information level as other more complex indicators [39]. Here, the PLV is used to analyze the phase synchronization between two channels of EEG in specific frequency, defined as follow:

(1) $$\begin{array}{cc} PLV_{n} & =\frac{1}{N}\left|\sum_{k=1}^{N}e^{i(\phi (t,k)-\psi (t,k))}\right| \end{array}$$

where *N* represent the number of epochs, ϕ(t,k) and ψ(t,k) indicates the phase values of channel ϕ and ψ for the epoch *n* at the time *t*. Specifically, we used HERMES toolbox [40] to obtain PLV matrices. In our case, 12 channels of EEG were used, which resulted in 12 × 12 symmetric matrix for each epoch. Each entry in matrix stood for synchronization of a pair of channels. This synchronization calculation was done for six frequency bands of each subject. The one-way ANOVA was used to assess differences between sleep stages or frequency bands of the PLV for REM, N2 and N3 stages. The observed returning values of ANOVA is *p*-value and lower *p*-value means more significant difference.

In addition, we also compared the brain network analysis between sleep stages with PLV matrices. We averaged the PLV matrix for each sleep stage and constructed the brain networks based on a threshold. The threshold is selected from the maximum value at which no isolated points appearing in the network.

#### 2.2.3. Band Evaluation

We use bi-serial correlation coefficient as an indicator to measure the ability of features to classify classes. The bi-serial correlation coefficient, $r^{2}$, is a measurement to evaluate the performance of one feature in distinguishing various classes. For a two-classes classification scenario (class *1*, 2), the bi-serial correlation coefficient is defined as:

(2) $$r_{X}^{2}={\left[\frac{\sqrt{N^{+}\times N^{-}}\times \left[mean\left(X^{+}\right)-mean\left(X^{-}\right)\right]}{\left(N^{+}\times N^{-}\right)\times std\left(X^{+}\cup X^{-}\right)}\right]}^{2}$$

where $X^{+}$ and $X^{-}$ represent all samples of class 1 and class 2, respectively. The $N^{+}$ and $N^{-}$ indicate the number of two class samples [41]. The $r^{2}$ is ranging from 0 to 1 and its bigger value means more discriminate between the two classes. We calculated the bi-serial correlation coefficient between every two sleep stages (REM and N2, REM and N3, and N2 and N3) for PLV feature matrices. In total, we sorted the 3 × 66 × 6 (sleep stages × PLV × frequency bands) $r^{2}$ values to evaluate the features across different frequency bands. We also defined the discriminative ratio as setting a threshold *t* to represent the number of features, calculating the sum of the first sorted *t* $r^{2}$ between every two sleep stages and then obtaining the ratio of each frequency band. In our case, *t* is set to 36.

#### 2.2.4. Classifier

We adopted support vector machine (SVM) with the Gaussian kernel function, which is implemented in the LIBSVM library [42]. The way to achieve multi-class classification is used the One-against-one strategy. We evaluated classification performance in terms of accuracy for single frequency band and the three level strategies fusion between frequency bands. The 75% samples were used for model training and the remaining 25% samples were used as testing data.

#### 2.2.5. Frequency Band Fusion Strategy

**Figure 3:**
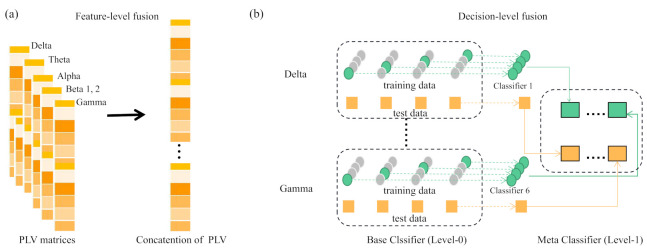
Schematic diagram of fusion strategy. (**a**) indicates the feature-level fusion and (**b**) is the decision-level fusion using stacking.

Based on the evaluation of brain functional connectivity across different frequency bands, we used three band fusion strategies to integration of multiple information sources for classification. The multiple information sources refer to the brain functional connectivity features of different frequency bands and the three fusion strategies are feature-level fusion, decision-level fusion, and hybrid-level fusion. The graphical description of feature-level and decision-level fusion strategies are shown in Figure 3.

For feature-level fusion, we concatenated the PLV features of six frequency bands before feeding into classifier; For decision fusion using stacking [43], we constructed individual classifiers for PLV features of a single frequency band separately, namely base classifier and then a meta classifier learns to use the predictions of each base classifier to obtain a target decision result. For hybrid fusion, we combined feature-level and decision-level fusions, which includes two steps: first constructing individual classifiers for specific groups of selected two frequency bands based on the band evaluation result and then conducting ensemble classifier of these individual classifiers. Hereinafter, ‘C’, ‘E’ and ’E(C)’ represent the classification result obtained by using the feature after feature-level fusion, decision-level fusion and hybrid fusion strategy, respectively.

## 3. Results

### 3.1. PLV Values between Six Frequency Bands for Different Sleep Stages

**Figure 4:**
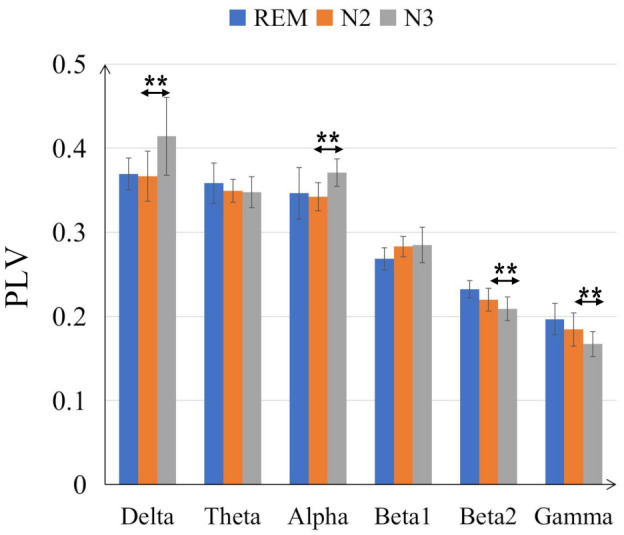
Comparisons of the average PLV values between six frequency bands for three sleep stages. The double-asterisk ‘**’ indicates that there is a significant difference of *p* < 0.001 by ANOVA test.

**Figure 5:**
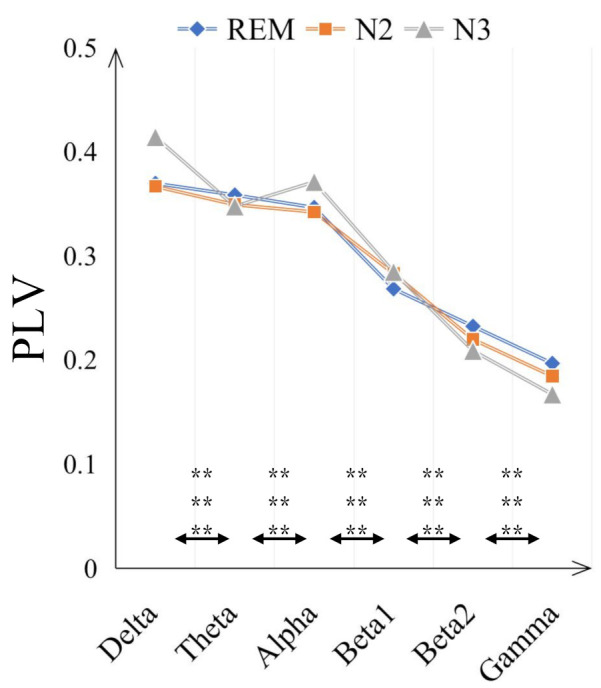
The averaged PLV value of three sleep stages in six frequency bands to observe the PLV distribution in different frequency bands. The double-asterisk ‘**’ indicates that there is a significant difference of *p* < 0.001 by ANOVA test and three rows represent the significant difference result between two frequency bands for ’REM and N2’, ’REM and N3’ and ’REM and N2’ respectively.

**Figure 6:**
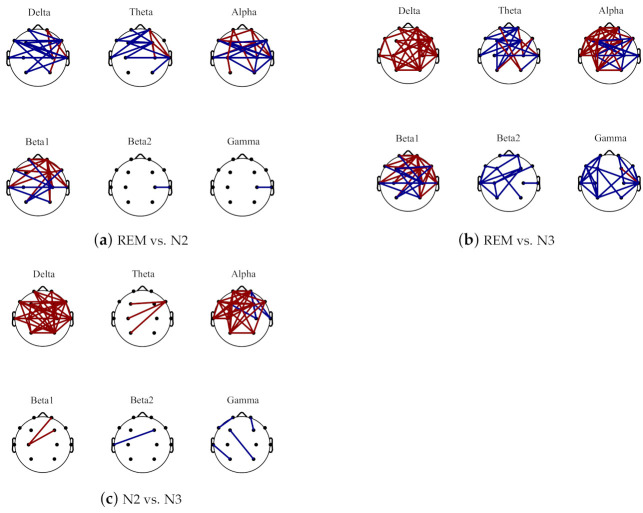
The brain network difference topoplot between REM and N2 (shown in (**a**)), REM and N3 (shown in (**b**)) and N2 and N3 (shown in (**c**)) for different frequency bands. The red line represents the increase area, and the blue line indicates the decrease area.

The comparisons on average PLV between six frequency bands for three sleep stages are in Figure 4. In some low frequency bands, delta and alpha bands, the PLV values are increased from N2 to N3 sleep stages while in high frequency bands, beta2 and gamma bands, the PLV values are decreased. Moreover, compared with N2 sleep stage, the PLV value for N3 is significantly bigger (with *p* < 0.001) in delta and alpha bands but smaller in beta 2 and gamma bands. Generally, the PLV values are significantly decreased as the frequency bands increase, from delta to gamma bands for REM, N2 and N3 sleep stages. Only the PLV value of alpha is significantly bigger than theta band, shown in Figure 5. Figure 6 displays the differences between sleep stages for brain network analysis from which the spatial distributions of PLV differences can be observed.

### 3.2. Evaluation of Different Frequency Bands

**Figure 7:**
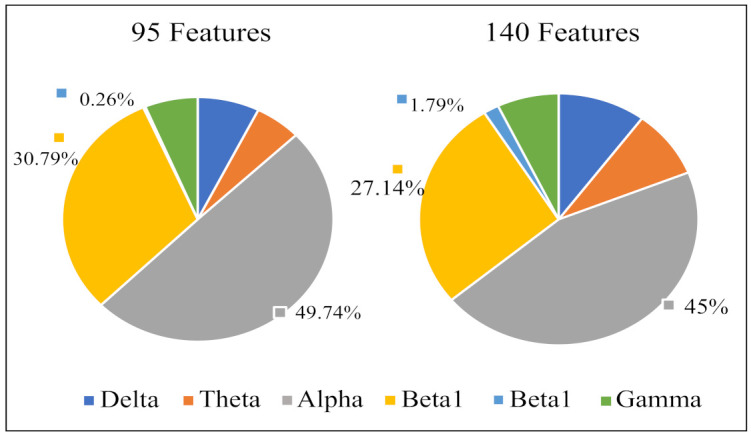
Percentage (%) of PLV features from different frequency bands in the features with first 95 and 140 sorted $r^{2}$ values.

**Figure 8:**
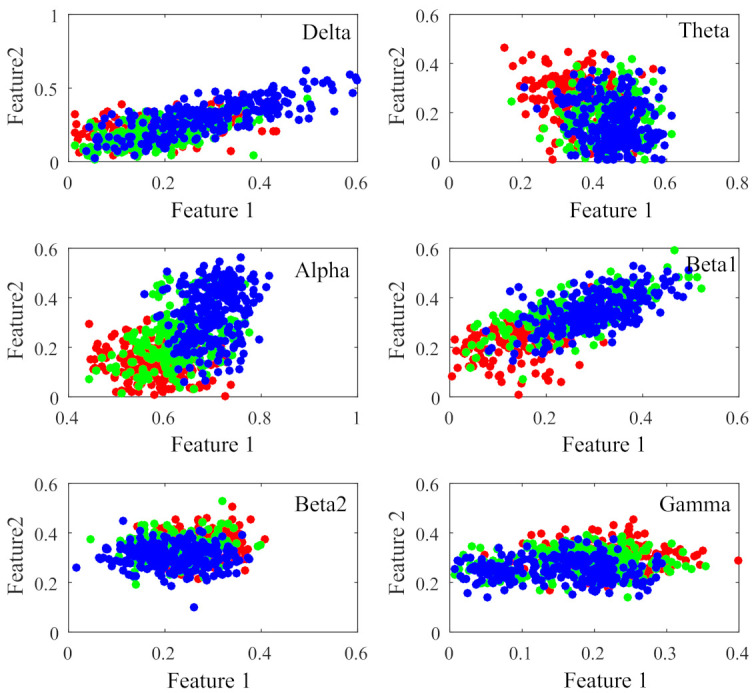
2-D figure of PLV feature visualization for each frequency band. The Feature 1 and Feature 2 are the selected features with the first two largest $\bar{r^{2}}$ values, respectively. The dots represent different sleep stages (red:REM, green:N2, blue:N3).

**Figure 9:**
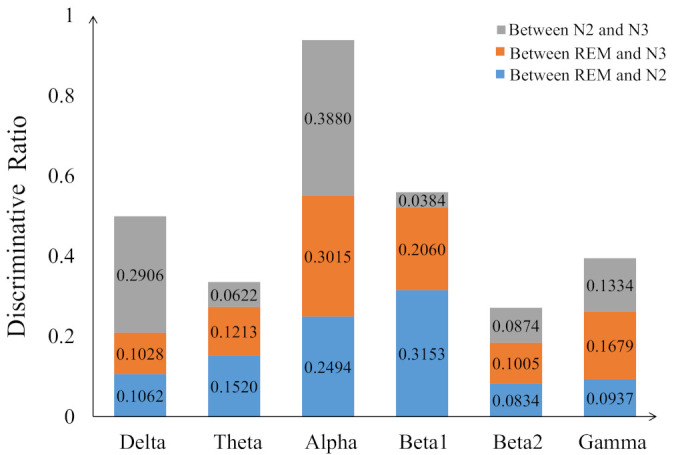
The discriminative ratio of per frequency band for distinguish two sleep stages (blue: REM and N2, orange: REM and N3, grey: N2 and N3).

Figure 7 displays the percentage of corresponding frequency bands within the first 95 and 140 sorted $r^{2}$ values cases. Alpha band shows the largest percentage in both cases, accounting for 49.74% and 45.00% and followed by beta1, accounting for 30.79% and 27.14%, respectively. The smallest percentage is beta2 band with only 0.26% and 1.79%. The 2-D figure of PLV feature visualization for each frequency band is shown in Figure 8. The ’Feature 1’ and ’Feature2’ are the features with first two largest $\bar{r^{2}}$ and the $\bar{r^{2}}$ is averaged through the three $r^{2}$ obtained between each two classes. We can see that the PLV features extracted from alpha band shows a better discriminate ability. Figure 9 reveals the discriminative ratio of frequency bands for sleep staging. Alpha band also shows the higher ratio than other bands which is consistent with the Figure 7 as well as the following Table 2. The discriminative ratios are different for a certain frequency band (such as delta and beta1 bands) in distinguishing different paired sleep stages. For instance, the ratio is not high in distinguishing REM and N2, REM and N3 while it reaches 29.06% in distinguishing N2 and N3.

### 3.3. Classification

#### 3.3.1. Classification Performance of Single-Band Feature

The average classification accuracy of PLV with single-band for intra-subject and inter-subject are shown in Table 2. For intra-subject case, alpha band outperforms other bands except for subject n5 and the best classification accuracy reaches 94.96% followed by beta1 band 92.81% (from subject n10). For inter-subject case, alpha band also outperforms other bands. The best classification accuracy reaches 90.86% followed by delta 86.86%; There is a special result that the accuracy of beta1 reaches 92.81% from subject n10, while the accuracy is only 82.86% in inter-subject case. The true positive rates for N2 and N3 are 76.57% and 81.07%, respectively and they are smaller than other bands.

#### 3.3.2. Classification Performance for Bands Fusion

Based on the results of different frequency bands evaluation (Section 3.2), we explored the three band fusion strategies for sleep staging. The two, three and four frequency bands are selected according to the $r^{2}$ values, respectively. Specifically, we combined ’delta and beta1’, ’theta and gamma’ and ’alpha and beta2’ since the delta band is complementary to beta1, the beta2 matches the best discriminative alpha, and theta and gamma are combined to make the three splicing results all good. For three frequency bands fusion case, the alpha, beta1 and delta frequency bands are selected according to Figure 7 and Figure 9 and further gamma frequency band is added to the four frequency bands fusion case. Finally, six frequency bands are used in feature-level, decision-level and hybrid-level fusions.

The classification performance of feature-level is better than decision-level and hybrid-level. Among the results of the fusion of six frequency bands, the best single subject is n3, with the accuracy rate of 96.91%, and the accuracy of inter-subjects is 96.14%. Note, compared with intra-subject and inter-subject cases, we can infer that there are not so big individual differences in sleep staging. The Table 3 shows detailed classification result.

## 4. Discussion

### 4.1. The Dominant Role of Alpha Band in Sleep Staging

The assessment of sleep staging is based on specific EEG frequencies and on the recognition of corresponding sleep-related EEG patterns [44]. For instance, the amount of alpha decreases and an increase of EEG in alpha range can be found during REM sleep while the alpha frequency is 1 to 2 Hz lower compared with wakefulness during non-REM sleep [45,46]. More Other related works also revealed that alpha band shows important role in sleep staging. Dkhil et al. [47] proposed the importance of alpha band in the evaluation of drowsiness. Knaut et al. [48] finds the changes in alpha oscillations reflect different brain states associated with different levels of wakefulness and thalamic activity. Specifically, in our study, alpha band shows dominant role in sleep staging since the PLV values are decreased as the frequency band increased, but alpha band is higher than theta band (see Figure 5); in the frequency band evaluation section (Section 3.2), alpha band shows higher discriminative ratio revealed by bi-serial correlation and 2-D feature topoplot also shows alpha band has the less overlapping area than other bands; for classification results (see Section 3.3), alpha band outperforms other bands in both intra-subject (except for subject n5) and inter-subject cases.

### 4.2. Inconsistency between Frequency Band Evaluation and the Classification Accuracy of Beta1 Band

EEG beta activity represents a marker of cortical arousal [49]. The conventional power spectrum shows a significant increase of EEG in beta band of patients with primary insomnia during N2 stage [50] while a decrease of patients with idiopathic REM sleep behavior disorder during phasic REM [51] compared with sleep health people. In our study, we observed the inconsistency between frequency band evaluation and the classification accuracy of EEG functional connectivity pattern in beta1 frequency band. In frequency band evaluation, the number of selected features of beta1 band is only less than alpha band (see Figure 7) and discriminative ratios of beta1 between ’REM and N2’ and ’REM and N3’ are very high, but the classification accuracy is generally low. We can also observe that the discriminative ratios of beta1 between ’N2 and N3’ is lowest in all bands (see Figure 9) and the true positive rate of the N2 and N3 stages is lower than other frequency bands (see Table 2). This may explain the inconsistency of the beta1 frequency band between band evaluation and classification accuracy. The special result from subject n10 shows achieved a high accuracy rate of 92.81% in beta1, while only 74.74–82.86% for other cases. Tracking the original data of subject n10, we found that there is no S3 stage which results in the deceasing decision error between N2 and N3.

### 4.3. Comparisons with Start-of-Arts Works

We compared our method with the start-of-arts in sleep staging classification research, shown in Table 4. Sors et al. [52] designed an end-to-end convolutional neural network(CNN) to classify the sleep stages using the raw signals and obtained the accuracy of 90.74%. Sharma et al. [53] used classical three time-domain features: log energy, signal fractal dimension, and signal sample entropy and multi-class SVM as the classifier and the ACC(N1 U N2, N3, REM) is 81.13%. Lajnef et al. [54] extracted 102 features covering the time-domain, frequency-domain and non-linear features. The accuracy of three classifications (REM, N2, N3) is 87.06% obtained by decision-tree-based multi-SVM; Michielli et al. [55] also combined feature extraction and deep learning and the ACC(REM U N1, N2, N3) is 90.6%. We also used their method on the CAP data set. Our method shows a better accuracy result compared with these related works. Hopefully, this method would be applied for automatic sleep staging and medical intervention of sleep disorders. For instance, in chronic insomnia, using transcranial direct current stimulation in N2 can increase the duration of N3 and sleep efficiency and the probability of transition from N2 to N3 [56] which requires more accurate separation of N2 and N3.

## 5. Conclusions

In this paper, we proposed a method to classify sleep stages with brain functional connectivity. We analyzed the characteristics of brain network during different sleep stages and explored the influences of frequency bands for sleep staging classification. After applying a simple machine learning method (multi-class SVM) and a series of analysis, we obtained that:

1. For brain functional connectivity values, the average PLV increases in the delta and alpha band, while decreases in the high frequency beta2 and gamma band during non-REM periods;
2. Different frequency bands have different discriminative abilities for distinguishing between sleep stages. Herein, alpha band show the dominant role in sleeping stage. Beta1 band shows good performance for classifying ’REM and N2’ and ’REM and N3’ but higher classification error rate for ’N2 and N3’.
3. The classification performance of PLV is better than state-of-art studies. The best accuracy is 96.91% and 96.14% for intra-subject and inter-subject cases, respectively. We also replicated time-domain, frequency-domain and non-linear features on the data set used in our paper and results show the better performance of PLV.
  In the future, we plan to develop on online brain computer interface for automatic sleep staging monitoring combined with this approach and graph convolution network.

## Figures and Tables

**Table 1:** Types and spatial distributions of brain waves during stages of sleep, ‘/’ means no appearance.

	N1/S1	N2/S2	N3		REM
			S3	S4	
delta waves	/	<20%	25∼50% 0.75∼3 Hz	>50%	/
alpha waves	<50%	/	/	/	mainly in Occipital lobe
sleep spindle waves	/	12.5∼15.5 Hz, occur in central, bilateral frontal, parietal, forehead, temporal lobes	about 12 Hz gradually reduce, mainly in frontal lobe	6–10 Hz, gradually disappear, mainly in frontal lobe	/
K-complex waves	/	occur mainly in frontal lobe	evoked by external stimuli	evoked by strong stimuli	/

**Table 2:** Classification accuracy (%) of PLV value with single-band. For inter-subject case, the true positive rate of each class is also given.

Band	n3	n5	n10	n11	Inter-Subjects			
					REM	N2	N3	ACC
delta	85.80	89.31	89.21	84.98	84.86	87.87	87.65	86.86
theta	82.10	85.53	74.82	82.16	86.24	81.87	87.24	84.86
alpha	88.27	86.79	**94.96**	88.73	93.58	88.70	90.53	**90.86**
beta1	76.54	74.74	**92.81**	82.63	91.74	**76.57**	**81.07**	82.86
beta2	85.80	79.25	87.05	78.87	81.19	80.75	86.01	82.71
gamma	82.72	75.47	76.98	85.92	85.78	79.50	85.19	83.43

**Table 3:** Classification accuracy (%) of PLV value with band fusion strategies. For inter-subject case, the true positive rate of each class is given.

		n3	n5	n10	n11	Inter-Subjects			
						REM	N2	N3	ACC
Two bands	C(delta+beta1)	87.65	93.08	91.37	91.08	92.20	88.70	88.48	89.71
	C(theta+gamma)	88.27	88.68	89.93	88.73	93.58	87.03	93.42	91.29
	C(alpha+beta2)	91.36	88.68	95.37	88.26	94.04	88.70	91.77	91.43
Three bands	C(alpha+beta1+delta)	94.41	93.08	92.81	94.37	93.12	92.05	95.06	93.43
	E(alpha+beta1+delta)	91.30	91.19	94.96	92.49	96.33	92.89	95.88	93.43
Four bands	C(alpha+beta1+ delta+gamma)	96.89	94.34	92.81	94.37	94.95	94.14	95.47	94.86
	E(alpha+beta1+ delta+gamma)	93.79	91.19	94.96	93.90	96.33	92.89	95.88	95.00
Six bands	Concatenation	**96.91**	95.60	94.24	96.71	96.33	94.98	97.12	**96.14**
	Ensemble	95.06	92.45	93.53	93.90	96.33	93.72	95.88	95.29
	E(C)	93.21	93.08	94.96	93.90	95.87	94.98	95.47	95.43

**Table 4:** Comparison of state-of-the-art studies.

Authors	Features	Database	Classifier	Results (%)			
				REM (+N1)	N2 (+N1)	N3	ACC
Sors et al. [52] 2018	raw signal samples	SSH-1	CNN	90.54	85.8	92.48	90.74
Sharma et al. [53] 2018	time-domain	Sleep-EDF	Multi-class SVM	71.81	82.57	84.48	81.13
		CAP	Multi-class SVM	84.40	84.10	85.60	84.71
Lajnef et al. [54] 2015	frequency-domain	DyCog Lab’PSD records	D-SVM	89.13	81.63	87.88	87.06
		CAP	Multi-class SVM	87.61	82.85	90.95	87.14
Michielli et al. [55] 2019	statistical features and spectral features	Sleep-EDF	LSTM -RNN	91.59	89.55	92.09	90.60
		CAP	Multi-class SVM	96.79	86.61	93.42	92.14
Proposed method	PLV	CAP	Multi-class SVM	96.33	94.98	97.12	**96.14**

## Data Availability

The data used in the manuscript is a public dataset, namely CAP Sleep Database, please see the link https://www.physionet.org/content/capslpdb/1.0.0/, accessed on 9 November 2018. We conform to the terms of the specified license.
